# Patient-reported outcomes for the Intergroup Sentinel Mamma study (INSEMA): A randomised trial with persistent impact of axillary surgery on arm and breast symptoms in patients with early breast cancer

**DOI:** 10.1016/j.eclinm.2022.101756

**Published:** 2022-11-25

**Authors:** Toralf Reimer, Angrit Stachs, Kristina Veselinovic, Silke Polata, Thomas Müller, Thorsten Kühn, Jörg Heil, Beyhan Ataseven, Roland Reitsamer, Guido Hildebrandt, Michael Knauer, Michael Golatta, Andrea Stefek, Dirk-Michael Zahm, Marc Thill, Valentina Nekljudova, David Krug, Sibylle Loibl, Bernd Gerber, INSEMA investigators

**Affiliations:** a Department of Obstetrics and Gynecology, University of Rostock, Südring 81, 18059 Rostock, Germany; b Department of Obstetrics and Gynecology, University of Ulm, Prittwitzstr. 43, 89075 Ulm, Germany; c Breast Center, Evangelisches Waldkrankenhaus Spandau, Stadtrandstr. 555, 13589 Berlin, Germany; d Women's Hospital, Klinikum Hanau GmbH, Leimenstr. 20, 63450 Hanau, Germany; e Women's Hospital, Klinikum Esslingen, Hirschlandstr. 97, 73730 Esslingen, Germany; f Breast Unit, University Hospital, University of Heidelberg, Im Neuenheimer Feld 460, 69120 Heidelberg, Germany; g Department of Gynecology and Gynecologic Oncology, Evang. Kliniken Essen-Mitte, Henricistr. 92, 45136 Essen, Germany; h Department of Obstetrics and Gynecology, LMU University Hospital, Marchioninistr. 15, 81377 Munich, Germany; i Breast Center, LKH Salzburg, Paracelsus Medical University Clinics, Müllner Hauptstr. 48, A-5020 Salzburg, Austria; j Department of Radiotherapy, University of Rostock, Südring 75, 18059 Rostock, Germany; k Brustzentrum Ost, Rohrschacher Str. 286, CH-9016 St. Gallen, Switzerland; l Breast Unit, Sankt Elisabeth Hospital, Max-Reger-Str. 5-7, 69121 Heidelberg, Germany; m Women's Hospital, Johanniter-Hospital Stendal, Wendstr. 31, 39576 Stendal, Germany; n Breast Center, SRH Waldklinikum Gera, Str. des Friedens 122, 07548 Gera, Germany; o Department of Gynecology and Gynecological Oncology, Agaplesion Markus Hospital, W.-Epstein-Str. 4, 60431 Frankfurt/Main, Germany; p German Breast Group, Dornhofstr. 10, 63263 Neu-Isenburg, Germany; q Department of Radiation Oncology, University Hospital Schleswig-Holstein, Arnold-Heller-Str., 24105 Kiel, Germany

**Keywords:** Axillary surgery, Breast cancer, Patient-reported outcomes, Quality of life, Sentinel lymph node biopsy

## Abstract

**Background:**

In clinically node-negative breast cancer patients, the INSEMA trial (NCT02466737) assessed the non-inferiority of avoiding sentinel lymph node biopsy (SLNB) or axillary lymph node dissection (ALND). Here we present patient-reported outcomes (PROs) as a secondary endpoint.

**Methods:**

PROs were assessed for patients with no axillary surgery, SLNB alone, and ALND. Quality of life (QoL) questionnaire EORTC QLQ-C30 and its breast cancer module (BR23) were used at baseline (pre-surgery) and 1, 3, 6, 12, and 18 months after surgery. The QoL scores were compared using repeated measures mixed models based on the safety set.

**Findings:**

Between 2015 and 2019, 5502 patients were recruited for the first randomization, and 5154 were included in the intent-to-treat set (4124 SLNB versus 1030 no SLNB). In the case of one to three macrometastases after SLNB, 485 patients underwent second randomization (242 SLNB alone versus 243 ALND). Questionnaire completion response remained high throughout the trial: over 70% at all time points for the first randomization. There were significant differences for the BRBS (breast symptoms) and BRAS (arm symptoms) scores favoring the no SLNB group in all post-baseline assessments. Patients in the SLNB group showed significantly and clinically relevant higher scores for BRAS (differences in mean values ≥5.0 points at all times), including pain, arm swelling, and impaired mobility in all postoperative visits, with the highest difference at one month after surgery. Scoring of the QLQ-C30 questionnaire revealed no relevant differences between the treatment groups, although some comparisons were statistically significant.

**Interpretation:**

This is one of the first randomized trials investigating the omission of SLNB in clinically node-negative patients and the first to report comprehensive QoL data. Patients with no SLNB benefitted regarding arm symptoms/functioning, while no relevant differences in other scales were seen.

**Funding:**

Supported by German Cancer Aid (Deutsche Krebshilfe, Bonn, Germany), Grant No. 110580 and Grant No. 70110580 to University Medicine Rostock.

**Research in context**

**Evidence before this study**

It is assumed that reduced axillary intervention leads to improved quality of life (QoL), although there is only indirect level I evidence supporting this assumption to date. Three studies, including 1755 patients, reported QoL for the setting axillary sentinel lymph node biopsy (SLNB) versus complete axillary lymph node dissection (ALND), of which two trials found improved QoL after SLNB. The IBCSG 10-93 trial randomized women aged 60 years and older to ALND versus no ALND. However, these trials do not provide evidence concerning QoL outcomes for patients who undergo axillary SLNB versus no axillary intervention.

**Added value of this study**

We report for the first time comprehensive QoL data using patient-reported outcomes (PROs) for patients with and without SLNB (Rando 1) and for SLNB-positive patients with and without completion ALND (Rando 2). Over a follow-up period of 18 months after surgery, arm symptoms with significant differences and breast symptoms with a tendency were improved in de-escalated treatment arms.

**Implications of all the available evidence**

While the surgical community is waiting for oncologic outcomes of the SOUND and INSEMA trials, our report supports the current strategies for de-escalation of axillary surgery from a patient perspective in clinically node-negative patients and upfront breast-conserving surgery.

## Introduction

The publication of ACOSOG Z0011 trial outcomes^1^^,^^2^ opened a decade of de-escalation trials for axillary surgery in early breast cancer. The Intergroup-Sentinel-Mamma (INSEMA) trial (NCT02466737, GBG-75, ABCSG-43) investigates the non-inferiority of omission of axillary surgical staging versus sentinel lymph node biopsy (SLNB) in patients with clinically node-negative breast cancer (tumor size ≤5 cm) and upfront breast-conserving surgery (BCS) with invasive disease-free survival (iDFS) as the primary endpoint. In the case of pN1a(sn) in the SLNB arm, patients underwent a second randomization to complete axillary lymph node dissection (ALND) or no further axillary surgery.

Previously, we reported the characteristics of the first 1000 recruited INSEMA patients in 2017.^3^ Due to the frequent use of protocol-prohibited nodal fields in the ACOSOG Z0011 trial,^4^ a preplanned central quality assurance review process for radiotherapy planning was included in the INSEMA protocol, and the associated findings were published in 2020.^5^

It is assumed that reduced axillary intervention leads to improved quality of life (QoL), although there is only indirect level I evidence supporting this assumption to date. Three studies, including 1755 patients, reported QoL for the setting SLNB versus ALND, of which two trials found improved QoL after SLNB.^6^ Moreover, the IBCSG 10-93 trial randomized women aged 60 years and older to ALND versus no ALND and demonstrated that QoL was better in the group with no axillary surgery.^7^

However, these trials do not provide evidence concerning QoL outcomes for patients who undergo axillary SLNB versus no axillary intervention. Because QoL considerations are the primary motivation for abandoning SLNB, there is a need for randomized trials with QoL as a defined endpoint.^8^ Here we report for the first time comprehensive QoL data using patient-reported outcomes (PROs) for clinically node-negative patients with and without SLNB (Rando 1) and for SLNB-positive patients with and without completion ALND (Rando 2).

## Methods

### Patients

**Fig. 1:**
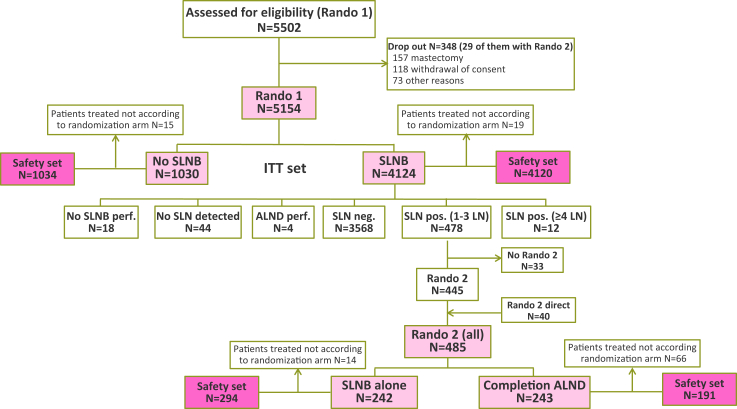
**CONSORT diagram**. Abbreviations: SLNB = sentinel lymph node biopsy; ITT = intention-to-treat; perf. = performed; SLN = sentinel lymph node; ALND = axillary lymph node dissection; neg. = negative; pos. = positive; LN = lymph node.

After approval by local independent review boards, INSEMA enrolled women ≥18 years old after informed consent between September 2015 and April 2019 in 142 German and nine Austrian study sites (Fig. 1). A total of 5502 patients were recruited for the first randomization (1:4 allocation, by Pocock minimization; randomization was stratified by age (<65 years vs. ≥65 years), tumor size (<2 cm vs. ≥2 cm), tumor grading (G1/2 vs. G3) and study site (German vs. Austrian, only for second randomization)), and 5154 of them were included in the intent-to-treat (ITT) set (N = 1030 with no SLNB versus N = 4124 with SLNB). The main reasons for a drop-out (5.8% for Rando 1) were secondary mastectomy and withdrawal of consent. In the second randomization, the drop-out rate was 6.0%, and 485 patients were recruited for the ITT set. The 1:1 distribution resulted in 242 women with SLNB alone and 243 women with completion ALND in case of one to three macrometastases in the SLNB. Short-term surgical complications were recorded over four weeks after final surgery for all patients by a physician, with documentation of lymphedema of the arm following diagnosis by the local investigator using site-specific definition.

All clinically node-negative patients with tumor size ≤5 cm underwent unilateral primary BCS with postoperative whole-breast irradiation (WBI) regardless of the intrinsic subtype. The use of high tangents or regional nodal irradiation was not permitted.^5^ A boost to the tumor bed was indicated for the majority of patients. The boost application could be omitted in cases at lower risk for local recurrence (age >60 years, small tumor size, and favorable tumor biology). The use of partial breast irradiation alone was not allowed.

### Questionnaires

PROs were assessed at baseline (pre-surgery) and 1, 3, 6, 12, and 18 months after final surgery using the European Organisation for Research and Treatment of Cancer Quality-of-Life Questionnaire (EORTC QLQ-C30) and its breast cancer (BR23) module.^9^^,^^10^ Higher scores of C30 and BR23 (range 0–100) indicate better functioning and global health status (GHS)/QoL or worse symptom severity, respectively. Score differences of ≥5.0 points were considered clinically meaningful, according to previous publications.^11^, ^12^, ^13^ The GHS/QoL, various Functioning, the arm (BRAS), and breast (BRBS) symptoms scales were targeted for analyses following the formulae provided in the scoring manual.^14^ The EORTC QLQ-C30 and BR23 have been translated and validated in German (https://qol.eortc.org/questionnaires/).

### Statistics

The QoL scores and surgery-related complications were analyzed based on the safety set (including patients whose lymph node surgical procedure was not according to the randomized arm as treated). Mean QoL scores per timepoint (±standard error of mean) were presented graphically. Repeated measures mixed-effects models including baseline value as a covariate were used for QoL scores leading to P-values for “treatment” and “time” and for interaction “treatment-by-time”. The analyses were performed using data available as of November 2, 2021, for each randomization and additionally in the subgroups by age (<65 years vs. ≥65 years) and by BMI (<30 kg/m^2^ vs. ≥30 kg/m^2^). Categorical parameters (patient and tumor characteristics and surgical complications) were presented as absolute numbers and (valid) percent, surgical complications were compared between arms using Fisher's exact test. Analyses were performed using SAS® (Statistical Analysis Software; Cary, NC, USA) version 9.4 with SAS Enterprise Guide 8.3. All tests were two-sided, and P-values less than 0.05 were considered statistically significant.

### Role of the funding source

The funding source had no involvement in the study design, the collection, analysis, and interpretation of data, the report's writing, and the decision to submit the article for publication.

## Results

### Patient characteristics

Patient and tumor characteristics for the safety sets concerning randomization are presented in Table 1. All baseline parameters were well-balanced between treatment arms. The median age at diagnosis was 62.0 years (range 24.0–89.0). Most patients presented with low-risk breast carcinoma (78.6% pT1 stage, 98.5% hormone-receptor positivity, 3.6% HER2 positivity, and 3.6% G3 tumors). The majority (73.3%) had an invasive carcinoma of no special type (72.7% in SLNB vs. 75.8% in no SLNB arm), and 87.1% had Ki-67 values ≤20%.

**Table 1:** Patient and tumor characteristics concerning randomization (safety set).

Parameter	Category	Randomization 1		Randomization 2	
		SLNB N (%)	No SLNB N (%)	cALND N (%)	SLNB alone N (%)
Age	<65 years	2516 (61.1)	621 (60.1)	129 (67.5)	189 (64.3)
	≥65 years	1604 (38.9)	413 (39.9)	62 (32.5)	105 (35.7)
BMI	<30 kg/m^2^	3082 (74.8)	781 (75.6)	133 (69.6)	215 (73.1)
	≥30 kg/m^2^	1038 (25.2)	252 (24.49)	58 (30.4)	79 (26.9)
	Missing	0	1	0	0
pT stage	pT0/is/X	38 (0.9)	6 (0.6)	0	1 (0.3)
	pT1	3227 (78.3)	822 (79.5)	113 (59.2)	171 (58.2)
	pT2	826 (20.0)	200 (19.3)	76 (39.8)	118 (40.1)
	pT3/4	29 (0.8)	6 (0.6)	2 (1.0)	(1.4)
ER/PgR status	Negative	63 (1.5)	16 (1.5)	2 (1.0)	6 (2.0)
	Positive	4054 (98.5)	1017 (98.5)	189 (99.0)	288 (98.0)
	Missing	3	1	0	0
HER2 status	Negative	3968 (96.6)	986 (95.7)	182 (95.3)	279 (95.2)
	Positive	141 (3.4)	44 (4.3)	9 (4.7)	14 (4.8)
	Missing	11	4	0	1
Tumor grading	G1	1536 (37.3)	399 (38.6)	54 (28.3)	87 (29.6)
	G2	2438 (59.2)	595 (57.5)	128 (67.0)	193 (65.6)
	G3	146 (3.5)	40 (3.9)	9 (4.7)	14 (4.8)
Ki-67	≤20%	3406 (86.9)	858 (87.9)	148 (78.3)	243 (85.9)
	>20%	514 (13.1)	118 (12.1)	41 (21.7)	40 (14.1)
	Missing	200	58	2	11
R0 resection in primary BCS	No	335 (8.1)	81 (7.8)	30 (15.7)	44 (15.0)
	Yes	3785 (91.9)	953 (92.2)	161 (84.3)	250 (85.0)
Number of removed axillary LNs	Mean	3.0	0	15.1	2.5
	Median	2.0	0	15.0	2.0
	Missing	20	0	0	0
Breast reconstruction performed	No	3949 (97.2)	985 (96.9)	180 (95.7)	272 (97.8)
	Yes	112 (2.8)	32 (3.1)	8 (4.3)	6 (2.2)
	Missing	4	5	0	1

Abbreviations: cALND = completion axillary lymph node dissection; BMI = body mass index; ER = estrogen receptor; PgR = progesterone receptor; BCS = breast-conserving surgery; LNs = lymph nodes.

Patients who were not treated according to the protocol (N = 34 for the first randomization; N = 80 for the second randomization) were included in the QoL analysis according to the respective surgical procedure. This scenario relates more to the Rando 2 group, where 66 patients did not undergo completion ALND leading to an asymmetric distribution for the QoL analysis: N = 191 with completion ALND versus N = 294 with SLNB alone.

No differences in the application of postoperative systemic treatment were observed between randomization groups except for chemotherapy among Rando 1 population. The ignorance of nodal status did not lead to increased chemotherapy indications: adjuvant chemotherapy was prescribed for 108 patients (10.7%) with no SLNB versus for 533 patients (13.2%) in the SLNB group (P = 0.03). All QoL baseline parameters regarding GHS, functional scales, and symptom scales/items were well-balanced between arms (total N = 4120 SLNB versus N = 1034 no SLNB as treated; 207 patients received completion ALND after SLNB).

### Questionnaire completion

Questionnaire completion response remained high throughout the trial: over 70% at all time points for the first randomization and over 60% at all time points for the second randomization (Supplement Figure S1). The highest completion rates were observed for baseline questionnaires with 91.5% (Rando 1) and 89.7% (Rando 2), respectively. In the first randomization cohort 3921 (76.1%) questionnaires were returned at 1 month after final surgery, 3940 (76.4%) at 3 months, 4028 (78.2%) at 6 months, 3912 (75.9%) at 12 months, and 3655 (70.9%) at 18 months with no difference between treatment arms at all time points. The postoperative response rates were slightly lower for the second randomization: 328 (67.6%) at 1 month, 331 (68.2%) at 3 months, 342 (70.5%) at 6 months, 332 (68.5%) at 12 months, and 300 (61.9%) at 18 months. The response rate was higher in the ALND cohort compared with SLNB alone cohort at all time points.

### General QoL

The Supplement Figure S2A–G shows the mean scores of GHS, Functioning scales (Physical, Role, Emotional, Cognitive, and Social Functioning), and Body Image at different time points during the trial for the first randomization. Except for Emotional Functioning, all mean scores were lower at 1, 3, and 6 months after surgery than at baseline for both groups (SLNB versus no SLNB). Mean scores for Emotional Functioning were higher than baseline by all postoperative visits without any differences between treatment arms (P = 0.78). GHS mean scores recovered nearly to baseline at 12 and 18 months after surgery. The most significant decline from baseline was detected in Role Functioning scores at 1 and 3 months postoperatively with slow recovery over time, but still below the baseline. No tendency for recovery was observed for Cognitive Functioning; patients in both groups had no improvement at 12 and 18 months compared with previous visits. There were significant differences between treatment groups favoring no SLNB group for GHS (P < 0.001), Physical Functioning (P < 0.001), and Role Functioning (P < 0.001). Except for Role Functioning at one month, all observed differences were <5.0 points at all time points, so that described statistically significant differences did not lead to clinically meaningful differences.

The curves were slightly different when considering the second randomization subgroup (Supplement Figure S3A–G). Borderline clinically meaningful differences were observed for GHS at one month and Body Image at 6, 12, and 18 months after surgery, both scales favoring SLNB alone arm.

### Arm morbidity

**Fig. 2:**
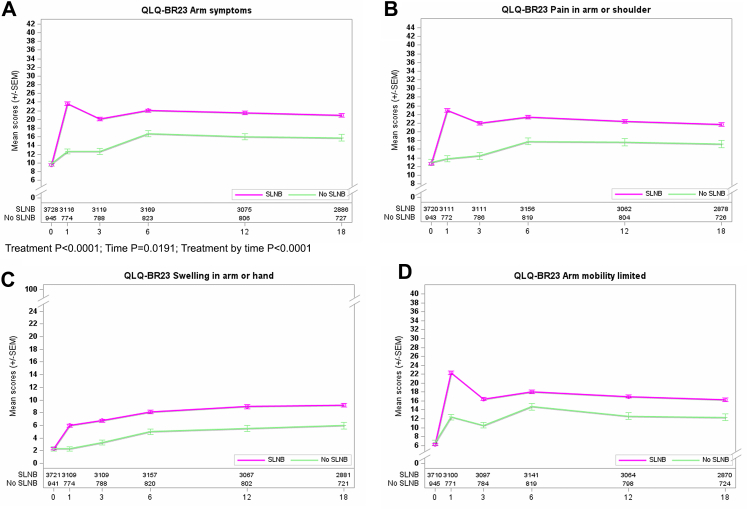
**Curves for PROs concerning QLQ-BR23 arm symptoms (BRAS scales) and first randomization**. A: overall with P-values for Treatment, Time, and Treatment by time; B: pain in arm or shoulder; C: swelling in arm or hand; and D: arm mobility limited.

The 3-item arm symptoms scores showed major differences between the first randomization treatment arms. Arm functioning worsened in both groups, but the deterioration was much greater in the SLNB group than in the no SLNB group (P < 0.001). The BRAS scores favor the no SLNB group in all post-baseline assessments (Fig. 2A). Patients in the SLNB group showed persistent higher scores for BRAS (differences in mean values ≥5.0 points at all times of evaluation), including pain, arm swelling, and impaired mobility in all postoperative visits, with the highest difference at one month after the final surgery (mean scores, 23.6 vs. 12.6). Fig. 2B–D shows the score curves for each BRAS item among the first randomization. The most remarkable differences between treatment arms were observed for pain in the arm or shoulder and limited arm mobility at one month postoperatively. Each item, including arm swelling, showed a persistent impact by treatment arms at all time points favoring the no SLNB group.

**Fig. 3:**
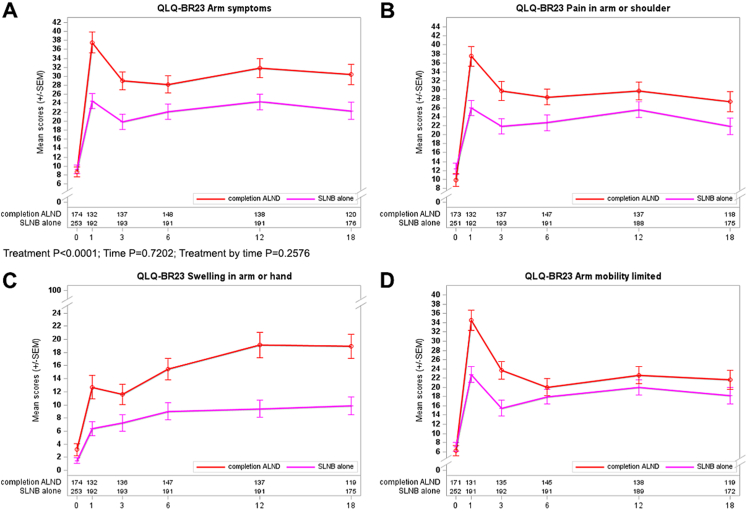
**Curves for PROs concerning QLQ-BR23 arm symptoms (BRAS scale) and second randomization**. A: overall with P-values for Treatment, Time, and Treatment by time; B: pain in arm or shoulder; C: swelling in arm or hand; and D: arm mobility limited.

The second randomization with a greater extent of axillary surgery revealed more significant differences using BRAS mean scores favoring SLNB alone at all post-baseline visits (P < 0.001). The prominent impairment occurred between 1 and 3 months following surgery (Fig. 3A). Separated analyses for each item showed clinically meaningful differences in pain in the arm or shoulder and swelling in the arm or hand at all time points (Fig. 3B–C). The worsening of arm swelling increased over time with major differences at 12 (mean values: 9.4 versus 19.2) and 18 months after surgery (mean values: 9.9 versus 18.9). Arm mobility was significantly more restricted in the completion ALND arm between 1 and 3 months postoperatively with the alignment of the curves between 6 and 18 months (Fig. 3D).

### Breast morbidity

**Fig. 4:**
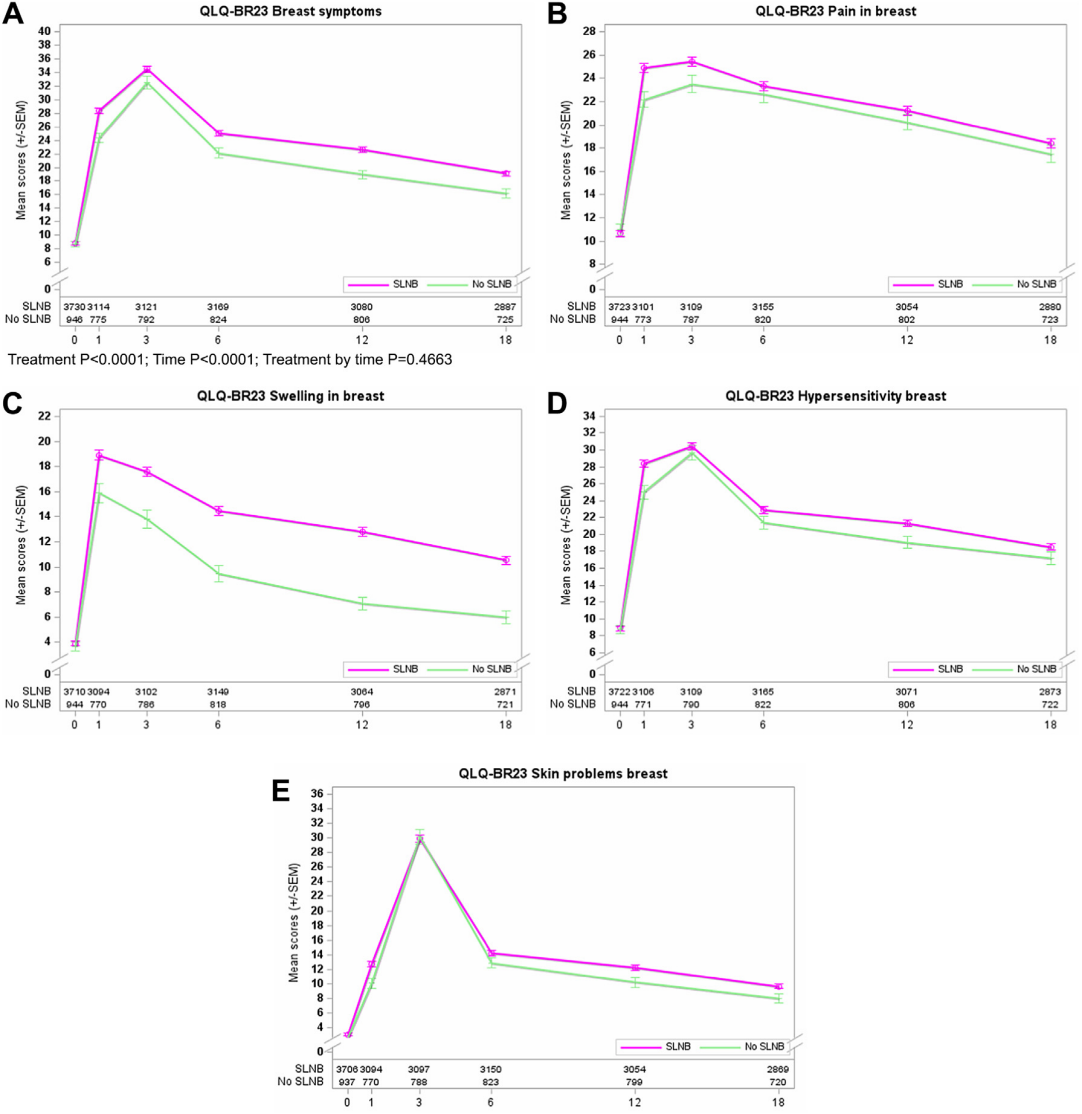
**Curves for PROs concerning QLQ-BR23 breast symptoms (BRBS scales) and first randomization**. A: overall with P-values for Treatment, Time, and Treatment by time; B: pain in the breast; C: swelling in the breast; D: hypersensitivity breast; and E: other skin problems in the breast.

The 4-item breast symptoms score showed statistically significant differences between treatment arms (SLNB versus no SLNB, P < 0.001) at all post-baseline assessments. However, this separation of the curves was not clinically relevant due to mean score differences of less than 5.0 points at all postoperative time points (Fig. 4A). In contrast to arm symptoms, breast symptoms significantly worsened in both groups over the first six months after surgery, followed by a slow recovery between 12 and 18 months. Single analysis of each BRBS item, including pain, breast swelling, hypersensitivity, and other skin problems, showed a smaller range but still a continuous trend for improved QoL in the no SLNB arm (Fig. 4B–E). Clinically meaningful differences were observed for the symptom ‘swelling in the breast’ between 6 and 18 months during longer follow-up.

**Fig. 5:**
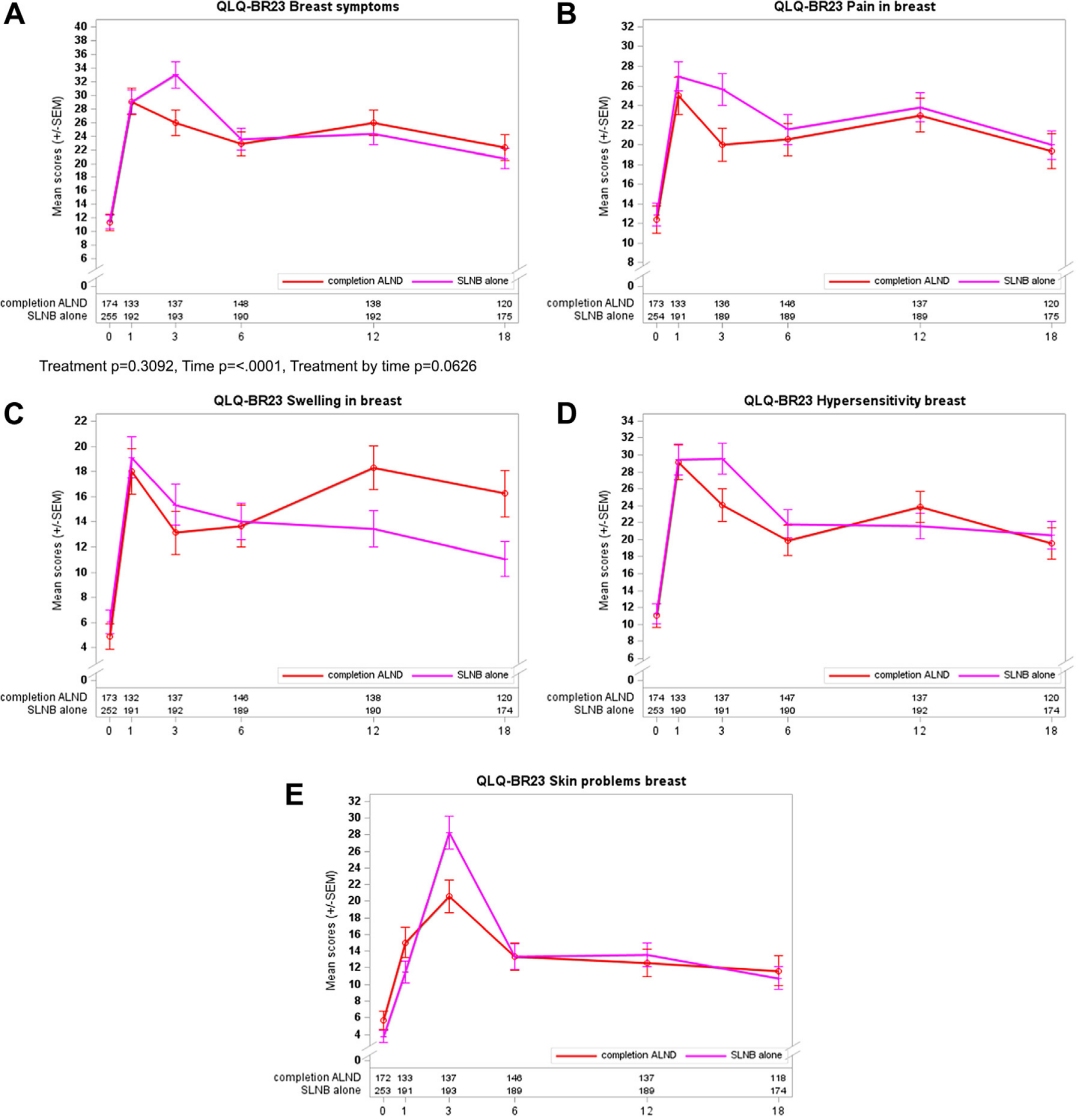
**Curves for PROs concerning QLQ-BR23 breast symptoms (BRBS scales) and second randomization**. A: overall with P-values for Treatment, Time, and Treatment by time; B: pain in the breast; C: swelling in the breast; D: hypersensitivity breast; and E: other skin problems in the breast.

The global breast symptoms score revealed no statistical difference between the treatment arm of the second randomization (Fig. 5A; P = 0.295). Considering single breast symptoms, there is a trend of less morbidity three months after surgery for all items favoring completion ALND. During longer follow-up, the item ‘swelling in the breast’ showed a significant advantage for SLNB alone arm with the highest differences at 18 months after surgery (mean scores 11.1 versus 16.3; Fig. 5B–E).

### Factors influencing BRAS and BRBS analyses

Completion of ALND among the standard SLNB group did not change the results of BRAS and BRBS score analyses. The curves’ patterns (shown in Fig. 2, Fig. 4A and 4A) were identical for the Rando 1 population irrespective of completion ALND in 207 patients.

Differences between treatment arms (Rando 1) for BRAS mean scores were more pronounced in the younger age group (<65 years) when compared to older patients (≥65 years) at 1 and 3 months. An inverse correlation was observed for BRBS analysis (Rando 1), showing more visible differences in the older age subgroup over the entire follow-up period. Age-dependent study for Rando 2 is limited due to smaller case numbers, but age (<65 versus ≥65 years) showed no impact on arm or breast symptoms.

Comparison of treatment arms concerning body mass index (BMI <30 kg/m^2^ versus ≥30 kg/m^2^) revealed more apparent differences among the BMI-high subgroup for arm symptoms. BMI did not affect breast symptoms (BRBS scale), focusing on the first randomization. The divergence of the BRAS curves was mainly observed between 3 and 6 months for the first randomization and between 3 and 12 months for the second randomization. In contrast, patients with BMI ≥30 kg/m^2^ recruited for the second randomization showed higher mean BRBS scores for completion ALND between 6 and 18 months after surgery. This negative impact of more radical axillary surgery on breast symptoms was not seen for the BMI-low subgroup.

### Short-term surgical complications

The rate of any postoperative surgery-related complications (Supplement Table S1) was significantly different between the first randomization arms (24.5% for the SLNB group versus 17.4% without SLNB; P < 0.001). Major differences were observed for seromas in the breast and/or axilla (6.1% versus 4.3%; P < 0.034), arm/shoulder mobility restriction (6.2% versus 2.8%; P < 0.001) or pain (7.3% versus 3.7%; P < 0.001), and paresthesias (3.4% versus 1.4%; P < 0.001).

The documented surgical complications (Supplement Table S1) were numerically higher among second randomization (30.9% with completion ALND versus 22.9% for SLNB alone); but it was not statistically significant (P = 0.06). Significant differences were only observed for lymphedema (3.5% versus 0.4%; P = 0.019) and arm/shoulder mobility restriction (10.0% versus 3.8%; P = 0.01).

## Discussion

Together with the Italian SOUND^15^ and the ongoing Dutch BOOG 2013-08^16^ trials, INSEMA is one of the first randomized trials investigating the omission of standard SLNB in clinically node-negative patients with early breast cancer and BCS. Additionally, INSEMA is one of four European ACOSOG Z0011 validation trials, including patients with one to three macrometastases in the SLNB.^17^ For the first time, we report comprehensive QoL data using PROs in both settings. PROs are a powerful tool for oncology care keeping in mind that a patient's experience is inadequately captured by physician documentation, particularly regarding symptoms.^18^

Previous data regarding side effects or impact on physical function and symptoms were published for the SOUND and ACOSOG Z0011 trials. Gentilini et al. reported the first 180 recruited SOUND patients using the QuickDASH (Disability Arm and Shoulder) questionnaire for the assessment of the physical function of the upper limb as a secondary outcome. Patients who underwent SLNB had a higher score of disability in the early postoperative period (one week after surgery) compared to patients with no SLNB. After 6 and 12 months, both groups’ scores decreased to values similar to baseline. Patients with completion ALND (N = 5) had a persistently higher rate of disability over the entire follow-up period.^19^

The ACOSOG Z0011 investigators reported significantly more wound infections, seromas, and paresthesias among patients with completion ALND (N = 399) than in the SLNB alone arm (N = 411). At one year of follow-up, lymphedema was reported by 13% of patients after completion ALND and 2% after SLNB alone.^20^ However, objective arm measurements did not confirm this significant difference regarding lymphedema. This gap in clinician-reported versus patient-reported symptoms is accepted since evaluations of the PRO-CTCAE (Common Terminology Criteria of Adverse Events) showed that patients grade symptoms up to 40% higher than clinicians.^21^ Physician-documented lymphedema rate was significantly higher in the completion ALND arm (3.5%) compared with SLNB arm within INSEMA trial, but with limited informative value due to short follow-up period (four weeks postoperatively).

Recently, the SENOMAC trialists published PROs for the first 1181 patients, including health-related QoL and arm morbidity, using EORTC QLQ-C30, QLQ-BR23, and Lymph-ICF (Lymphedema Functioning Disability and Health) questionnaires. The SENOMAC design is comparable with the second randomization of INSEMA but additionally allowed recruitment of patients with mastectomy (33.9% in both arms) and T3 tumors. Patients receiving SLNB alone reported significantly lower symptom scores on the EORTC subscales of pain, BRAS, and BRBS in the early postoperative phase and at one-year follow-up. The Lymph-ICF domain scores of physical function, mental function, and mobility activities were significantly in favor of the SLNB alone group.^22^

Interestingly, QoL analyses for SENOMAC and INSEMA showed identical results. No significant group differences in overall health-related QoL/GHS were identified in both trials. Most clinically meaningful differences were observed in arm morbidity-related items with no recovery after 12 months or 18 months after surgery. Unexpectedly, clinically relevant arm symptoms also occurred in the no SLNB arm within the INSEMA study. The score changes compared to the baseline survey were significantly less pronounced than in the SLNB arm, but persisted over the entire follow-up period. Thus, breast surgery without axillary intervention followed by radiotherapy also seems to have a moderate influence on arm symptoms on the affected side. Assuming ≥80% of prescribed breast dose as the optimal dose for curative radiation of low-volume disease in axillary lymph nodes, at least 50% of centrally reviewed INSEMA patients received an adequate dose in axillary level I, even with contemporary 3D techniques.^5^

This report presents the first QoL data for the complete omission of axillary surgery after enrolment in the INSEMA trial (at least 18 months follow-up after last-patient-in). Our results demonstrate a significant increase in arm and breast symptoms after SLNB compared to no SLNB. While the surgical community is waiting for oncologic outcomes of the SOUND and INSEMA trials, our report supports the current strategies for de-escalation of axillary surgery from a patient perspective.

So far, reported data, including the INSEMA trial, reported QoL data based on planned analysis of a secondary endpoint. Randomized data on PROs regarding arm morbidity and GHS/QoL as primary outcomes are scarce. The ALMANAC trial significantly reduced arm morbidity and improved QoL among patients randomized to SLNB versus standard ALND.^23^ Addenbrookes-2 investigators reported that QoL scores were higher in the SLNB group than in the ALND group for one year postoperatively.^24^ In contrast, the GIVOM Sentinella trial showed no significant differences between SLNB and ALND groups on the physical and health-related QoL components of the Short Form (SF)-36 measure.^25^ However, these trials with QoL-related primary endpoint provide no evidence regarding the complete omission of SLNB. Only the IBCSG 10-93 trial showed QoL data for a treatment arm with no axillary surgery in an elderly population.^7^

This INSEMA analysis has numerous strengths. Firstly, data are based on fully recruited cohorts so that INSEMA covers the largest population with available PROs in the setting of complete omission of axillary surgery in clinically node-negative patients. Secondly, the high questionnaire response rates allow representative results. Thirdly, a pre-surgery baseline assessment and a long follow-up period of 18 months after surgery are available for comprehensive analyses. Hamidou et al.^12^ address that, at 6 or 12 months postoperatively, patients could have recovered their baseline QoL level or shown a trend for QoL deterioration. Finally, previously published results regarding dose values of incidental axillary irradiation during postoperative WBI showed no differences between INSEMA treatment arms, ruling out a confounding impact on QoL scores.^5^

However, our study also has several limitations. Firstly, the analysis was restricted to EORTC QLQ-C30 and -BR23 questionnaires. A more detailed arm or breast morbidity questionnaire like Lymph-ICF was unavailable. Secondly, this secondary outcome analysis focuses on PROs. Clinician-reported symptoms (except short-term complications) or objective arm measurements were not evaluated. Long-term surgical adverse events will be reported together with primary outcome publication. Finally, this analysis reflects only the setting of upfront surgery in early breast cancer and should not be transferred to axillary surgery after neoadjuvant treatment.

In conclusion, QoL issues are particularly interesting in early breast cancer because effective methods for diagnostics and treatment have led to an increased number of long-term survivors.^11^ Over a follow-up period of 18 months after surgery, arm symptoms with significant differences and breast symptoms with a tendency, but not GHS/QoL, were improved in de-escalated treatment arms. These results are of high clinical relevance and support the ongoing discussion concerning the reduction or omission of axillary surgery in clinically node-negative patients and upfront BCS.

## Contributors

Toralf Reimer: (1) the conception and design of the study, accessed the raw data, acquisition of data, and analysis and interpretation of data, (2) drafting of the article, and (3) final approval of the submitted version.

Angrit Stachs: (1) the acquisition of data, (2) revising the article critically for important intellectual content, and (3) final approval of the submitted version.

Kristina Veselinovic: (1) the acquisition of data, (2) revising the article critically for important intellectual content, and (3) final approval of the submitted version.

Silke Polata: (1) the acquisition of data, (2) revising the article critically for important intellectual content, and (3) final approval of the submitted version.

Thomas Müller: (1) the acquisition of data, (2) revising the article critically for important intellectual content, and (3) final approval of the submitted version.

Thorsten Kühn: (1) the conception of the study and acquisition of data, (2) revising the article critically for important intellectual content, and (3) final approval of the submitted version.

Jörg Heil: (1) the acquisition of data, (2) revising the article critically for important intellectual content, and (3) final approval of the submitted version.

Beyhan Ataseven: (1) revising the article critically for important intellectual content, and (2) final approval of the submitted version.

Roland Reitsamer: (1) the acquisition of data, (2) revising the article critically for important intellectual content, and (3) final approval of the submitted version.

Guido Hildebrandt: (1) the conception and design of the study, acquisition of data, analysis, and interpretation of data, and (2) final approval of the submitted version.

Michael Knauer: (1) the conception of the study, (2) revising the article critically for important intellectual content, and (3) final approval of the submitted version.

Michael Golatta: (1) the acquisition of data, (2) revising the article critically for important intellectual content, and (3) final approval of the submitted version.

Andrea Stefek: (1) the acquisition of data, (2) revising the article critically for important intellectual content, and (3) final approval of the submitted version.

Dirk-Michael Zahm: (1) the acquisition of data, (2) revising the article critically for important intellectual content, and (3) final approval of the submitted version.

Marc Thill: (1) the acquisition of data, (2) revising the article critically for important intellectual content, and (3) final approval of the submitted version.

Valentina Nekljudova: (1) the conception and design of the study, accessed the raw data, analysis and interpretation of data, (2) revising the article critically for important intellectual content, and (3) final approval of the submitted version.

David Krug: (1) the interpretation of data, (2) revising the article critically for important intellectual content, and (3) final approval of the submitted version.

Sibylle Loibl: (1) the conception and design of the study, accessed the raw data, and interpretation of data, (2) revising the article critically for important intellectual content, and (3) final approval of the submitted version.

Bernd Gerber: (1) the conception and design of the study, accessed the raw data, acquisition of data, and analysis and interpretation of data, (2) drafting the article, and (3) final approval of the submitted version.

Toralf Reimer, Valentina Nekljudova, Sibylle Loibl, and Bernd Gerber have accessed and verified the data, and these authors were responsible for the decision to submit the manuscript. All authors approved the final article.

## Data sharing statement

All data generated and analyzed during this study are included in this published article.

## Declaration of interests

Toralf Reimer: Complete funding of the INSEMA trial by two grants from the German Cancer Aid (Deutsche Krebshilfe). All payments to the University Medicine Rostock (UMR), Germany. Grant from the Else Kroener-Fresenius Foundation for EUBREAST-01 trial; payment to the UMR. Grant from the German Society of Senology for EUBREAST-01 trial; payment to the UMR. Payment for presentations from Roche, Pfizer, AstraZeneca, Novartis, and Daiichi-Sankyo.

Angrit Stachs: no declared conflicts of interest.

Kristina Veselinovic: Payment or honoraria for presentations from Roche, Novartis, AstraZeneca, and Lilly.

Silke Polata: no declared conflicts of interest.

Thomas Müller: no declared conflicts of interest.

Thorsten Kühn: no declared conflicts of interest.

Jörg Heil: no declared conflicts of interest.

Beyhan Ataseven: no declared conflicts of interest.

Roland Reitsamer: no declared conflicts of interest.

Guido Hildebrandt: no declared conflicts of interest.

Michael Knauer: Participation of Data Safety Monitoring Board for WSG (Westdeutsche Studiengruppe, Germany). Leadership Swiss Society of Senology.

Michael Golatta: Grants/contracts from Siemens Healthcare GmbH and Samantree medical. Honoraria from Samantree medical for presentation (ESSO40). Advisory Board Medbotics (twice a year).

Andrea Stefek: no declared conflicts of interest.

Dirk-Michael Zahm: no declared conflicts of interest.

Marc Thill: Grants/contracts from Exact Sciences and Endomag, payment to my institution. Consulting fees from Agendia, Amgen, AstraZeneca, Becton and Dickinson, Clearcut, Daiichi Sankyo, Eisai, Exact Sciences, Gilead Sciences, Grünenthal, GSK, Lilly, Norgine, Neodynamics, Novartis, Onkowissen, Organon, Pfizer, Pfm medical, Pierre Fabre, Roche, RTI Surgical, Seagen, Sirius Pintuition, and Sysmex. Honoraria for presentations from Amgen, AstraZeneca, Connect Medica, Eisai, Exact Sciences, Gedeon Richter, Gilead Sciences, GSK, Hexal, I-Med-Institute, Joerg Eickeler, Lilly, MCI, Medscape, MSD, Medtronic, Novartis, Onkowissen, Pfizer, Pfm medical, Roche, Seagen, Streamed UP, Sysmex, Vifor, Viatris, and Servier. Support for attending meetings from Amgen, AstraZeneca, Celgene, Daiichi Sankyo, Hexal, Neodynamics, Clearcut, Novartis, Pfizer, Roche, Eisai, Exact Sciences, Art Tempi, Pfm medical, Roche, Hexal, MCI, Lilly, MSD, Norgine, Novartis, Pfizer, RTI Surgical, and Seagen. Leadership AWOgyn (chair of the board of directors) and DGGG (member of the board of directors). Receipt of equipment, materials, drugs, medical writing, gifts or other services from Roche, Servier, AstraZeneca, Celgene, RTI Surgical, Novartis, Amgen, Roche, Clearcut, and Pfm medical.

Valentina Nekljudova: Grants/contracts from Abbvie, AstraZeneca, BMS, Daiichi-Sankyo, Gilead, Novartis, Pfizer and Roche. Royalties or licenses from VM Scope GmbH. Patents planned, issued or pending: EP14153692.0, EP21152186.9, EP15702464.7, and EP19808852.8.

David Krug: no declared conflicts of interest.

Sibylle Loibl: Grants/contracts from Abbvie, AstraZeneca, DSI, Celgene, Gilead, Novartis, Pfizer, Roche, and Molecular H. Royalties or licenses: Digital Ki67 Evaluator. Honoraria for lectures from AstraZeneca, DSI, Gilead, Novartis, Pfizer, and Roche. Honoraria for medical writing from DSI, Gilead, Novartis, Pfizer, Roche, and Seagen. Patents planned, issued or pending: EP14153692.0, EP21152186.9, EP15702464.7, and EP19808852.8. Participation on a Data Safety Monitoring Board or Advisory Board: Abbvie, Amgen, AstraZeneca, BMS, Celgene, DSI, Eirgenix, Eisai Europe, GSK, Gilead, Lilly, Merck, Novartis, Pfizer, Pierre Fabre, Relay Therapeutics, Roche, Sanofi, and Seagen.

Bernd Gerber: no declared conflicts of interest.

## Appendix A Supplementary data

Supplementary Table S1 Clinician-reported surgical complications among the first randomization (SLNB versus no SLNB) observed within four weeks postoperatively (safety set).

Supplementary Table S2 Clinician-reported surgical complications among the second randomization (completion ALND versus SLNB alone) observed within four weeks postoperatively (safety set).

Supplementary Figure S1 Questionnaire response rates per follow-up timepoint with respect to the treatment arms.

Supplementary Figure S2 Curves for PROs concerning QLQ-C30 and QLQ-BR23 questionnaires and first randomization. According to a reference manual, reference lines are for stage I-II breast cancer, available only for QLQ-C30, not for QLQ-BR23 Body Image. A: Global Health Status; B: Physical Functioning; C: Role Functioning; D: Emotional Functioning; E: Cognitive Functioning; F: Social Functioning; and G: Body Image.

Supplementary Figure S3 Curves for PROs concerning QLQ-C30 and QLQ-BR23 questionnaires and second randomization. According to a reference manual, reference lines are for stage I-II breast cancer, available only for QLQ-C30, not for QLQ-BR23 Body Image. A: Global Health Status; B: Physical Functioning; C: Role Functioning; D: Emotional Functioning; E: Cognitive Functioning; F: Social Functioning; and G: Body Image.
