# Supplementary material for: Patient-reported outcomes for the Intergroup Sentinel Mamma study (INSEMA): A randomised trial with persistent impact of axillary surgery on arm and breast symptoms in patients with early breast cancer
Source: eClinicalMedicine. 2022 Nov 25;55:101756. doi: 10.1016/j.eclinm.2022.101756 (PMC9706517; doi:10.1016/j.eclinm.2022.101756)
Supplement: Supplementary Table S1 — Clinician-reported surgical complications among the first randomization (SLNB versus no SLNB) observed within four weeks postoperatively (safety set). [file mmc1.docx]

Supplement Tab.1: Clinician-reported surgical complications among first randomization (SLNB versus no SLNB) observed within four weeks postoperatively (safety set).

| **Parameter** | **Parameter value** | **SLNB** | **No SLNB** | **Overall** | **P-value** |
| --- | --- | --- | --- | --- | --- |
| Any surgical complication | no | 3058 (75.5) | 838 (82.6) | 3896 (76.9) | <.001 |
|  | yes | 993 (24.5) | 176 (17.4) | 1169 (23.1) |  |
|  |  |  |  |  |  |
|  | missing | 73 | 16 | 89 |  |
| Damage of vessels | no | 4032 (99.5) | 1013 (99.9) | 5045 (99.6) | 0.155 |
|  | yes | 19 ( 0.5) | 1 ( 0.1) | 20 ( 0.4) |  |
|  | missing | 73 | 16 | 89 |  |
| Damage of nerves | no | 4030 (99.5) | 1012 (99.8) | 5042 (99.5) | 0.292 |
|  | yes | 21 ( 0.5) | 2 ( 0.2) | 23 ( 0.5) |  |
|  | missing | 73 | 16 | 89 |  |
| Seromas in the breast and/or axilla | no | 3804 (93.9) | 970 (95.7) | 4774 (94.3) | 0.034 |
|  | yes | 247 ( 6.1) | 44 ( 4.3) | 291 ( 5.7) |  |
|  | missing | 73 | 16 | 89 |  |
| Haematomas | no | 3743 (92.4) | 938 (92.5) | 4681 (92.4) | 0.947 |
|  | yes | 308 ( 7.6) | 76 ( 7.5) | 384 ( 7.6) |  |
|  | missing | 73 | 16 | 89 |  |
| Lymphedema | no | 3986 (98.4) | 1004 (99.0) | 4990 (98.5) | 0.189 |
|  | yes | 65 ( 1.6) | 10 ( 1.0) | 75 ( 1.5) |  |
|  | missing | 73 | 16 | 89 |  |
| Wound infections | no | 3979 (98.2) | 1001 (98.7) | 4980 (98.3) | 0.338 |
|  | yes | 72 ( 1.8) | 13 ( 1.3) | 85 ( 1.7) |  |
|  | missing | 73 | 16 | 89 |  |
| Axillary web syndrome (cording) | no | 4031 (99.5) | 1013 (99.9) | 5044 (99.6) | 0.100 |
|  | yes | 20 ( 0.5) | 1 ( 0.1) | 21 ( 0.4) |  |
|  | missing | 73 | 16 | 89 |  |
| Arm or shoulder mobility restriction | no | 3799 (93.8) | 986 (97.2) | 4785 (94.5) | <.001 |
|  | yes | 252 ( 6.2) | 28 ( 2.8) | 280 ( 5.5) |  |
|  | missing | 73 | 16 | 89 |  |
| Arm or shoulder mobility pain | no | 3754 (92.7) | 976 (96.3) | 4730 (93.4) | <.001 |
|  | yes | 297 ( 7.3) | 38 ( 3.7) | 335 ( 6.6) |  |
|  | missing | 73 | 16 | 89 |  |
| Weakness | no | 3982 (98.3) | 992 (97.8) | 4974 (98.2) | 0.354 |
|  | yes | 69 ( 1.7) | 22 ( 2.2) | 91 ( 1.8) |  |
|  | missing | 73 | 16 | 89 |  |
| Pulmonary embolism | no | 4047 (99.9) | 1013 (99.9) | 5060 (99.9) | 1.000 |
|  | yes | 4 ( 0.1) | 1 ( 0.1) | 5 ( 0.1) |  |
|  | missing | 73 | 16 | 89 |  |
| Thrombosis | no | 4043 (99.8) | 1012 (99.8) | 5055 (99.8) | 1.000 |
|  | yes | 8 ( 0.2) | 2 ( 0.2) | 10 ( 0.2) |  |
|  | missing | 73 | 16 | 89 |  |
| Brachial plexus injury | no | 4046 (99.9) | 1013 (99.9) | 5059 (99.9) | 1.000 |
|  | yes | 5 ( 0.1) | 1 ( 0.1) | 6 ( 0.1) |  |
|  | missing | 73 | 16 | 89 |  |
| Paresthesias | no | 3912 (96.6) | 1000 (98.6) | 4912 (97.0) | <.001 |
|  | yes | 138 ( 3.4) | 14 ( 1.4) | 152 ( 3.0) |  |
|  | missing | 74 | 16 | 90 |  |
| Other | no | 3906 (96.4) | 979 (96.5) | 4885 (96.5) | 0.924 |
|  | yes | 144 ( 3.6) | 35 ( 3.5) | 179 ( 3.5) |  |
|  | missing | 74 | 16 | 90 |  |
| Not specified | no | 4040 (99.7) | 1012 (99.8) | 5052 (99.7) | 0.749 |
|  | yes | 12 ( 0.3) | 2 ( 0.2) | 14 ( 0.3) |  |
|  | missing | 72 | 16 | 88 |  |
