# Supplementary material for: Patient-reported outcomes for the Intergroup Sentinel Mamma study (INSEMA): A randomised trial with persistent impact of axillary surgery on arm and breast symptoms in patients with early breast cancer
Source: eClinicalMedicine. 2022 Nov 25;55:101756. doi: 10.1016/j.eclinm.2022.101756 (PMC9706517; doi:10.1016/j.eclinm.2022.101756)
Supplement: Supplementary Table S2 — Clinician-reported surgical complications among the second randomization (completion ALND versus SLNB alone) observed within four weeks postoperatively (safety set). [file mmc2.docx]

| Supplement Tab.2: Clinician-reported surgical complications among second randomization (completion ALND versus SLNB alone) observed within four weeks postoperatively (safety set). |
| --- |

| **Parameter** | **Parameter value** | **ALND** | **SLNB alone** | **Overall** | **P-value** |
| --- | --- | --- | --- | --- | --- |
| Any surgical complication | no | 159 (69.1) | 182 (77.1) | 341 (73.2) | 0.060 |
|  | yes | 71 (30.9) | 54 (22.9) | 125 (26.8) |  |
|  | missing | 13 | 6 | 19 |  |
| Damage of vessels | no | 229 (99.6) | 236 ( 100) | 465 (99.8) | 0.494 |
|  | yes | 1 ( 0.4) | 0 ( 0.0) | 1 ( 0.2) |  |
|  | missing | 13 | 6 | 19 |  |
| Damage of nerves | no | 228 (99.1) | 235 (99.6) | 463 (99.4) | 0.619 |
|  | yes | 2 ( 0.9) | 1 ( 0.4) | 3 ( 0.6) |  |
|  | missing | 13 | 6 | 19 |  |
| Seromas in the breast and/or axilla | no | 206 (89.6) | 215 (91.1) | 421 (90.3) | 0.639 |
|  | yes | 24 (10.4) | 21 ( 8.9) | 45 ( 9.7) |  |
|  | missing | 13 | 6 | 19 |  |
| Haematomas | no | 213 (92.6) | 216 (91.5) | 429 (92.1) | 0.733 |
|  | yes | 17 ( 7.4) | 20 ( 8.5) | 37 ( 7.9) |  |
|  | missing | 13 | 6 | 19 |  |
| Lymphedema | no | 222 (96.5) | 235 (99.6) | 457 (98.1) | 0.019 |
|  | yes | 8 ( 3.5) | 1 ( 0.4) | 9 ( 1.9) |  |
|  | missing | 13 | 6 | 19 |  |
| Wound infections | no | 222 (96.5) | 229 (97.0) | 451 (96.8) | 0.798 |
|  | yes | 8 ( 3.5) | 7 ( 3.0) | 15 ( 3.2) |  |
|  | missing | 13 | 6 | 19 |  |
| Axillary web syndrome (cording) | no | 228 (99.1) | 235 (99.6) | 463 (99.4) | 0.619 |
|  | yes | 2 ( 0.9) | 1 ( 0.4) | 3 ( 0.6) |  |
|  | missing | 13 | 6 | 19 |  |
| Arm or shoulder mobility restriction | no | 207 (90.0) | 227 (96.2) | 434 (93.1) | 0.010 |
|  | yes | 23 (10.0) | 9 ( 3.8) | 32 ( 6.9) |  |
|  | missing | 13 | 6 | 19 |  |
| Arm or shoulder mobility pain | no | 209 (90.9) | 223 (94.5) | 432 (92.7) | 0.155 |
|  | yes | 21 ( 9.1) | 13 ( 5.5) | 34 ( 7.3) |  |
|  | missing | 13 | 6 | 19 |  |
| Weakness | no | 225 (97.8) | 235 (99.6) | 460 (98.7) | 0.118 |
|  | yes | 5 ( 2.2) | 1 ( 0.4) | 6 ( 1.3) |  |
|  | missing | 13 | 6 | 19 |  |
| Pulmonary embolism | no | 230 ( 100) | 236 ( 100) | 466 ( 100) | n.a. |
|  | yes | 0 ( 0.0) | 0 ( 0.0) | 0 ( 0.0) |  |
|  | missing | 13 | 6 | 19 |  |
| Thrombosis | no | 229 (99.6) | 236 ( 100) | 465 (99.8) | 0.494 |
|  | yes | 1 ( 0.4) | 0 ( 0.0) | 1 ( 0.2) |  |
|  | missing | 13 | 6 | 19 |  |
| Brachial plexus injury | no | 230 ( 100) | 236 ( 100) | 466 ( 100) | n.a. |
|  | yes | 0 ( 0.0) | 0 ( 0.0) | 0 ( 0.0) |  |
|  | missing | 13 | 6 | 19 |  |
| Paresthesias | no | 219 (95.2) | 231 (97.9) | 450 (96.6) | 0.132 |
|  | yes | 11 ( 4.8) | 5 ( 2.1) | 16 ( 3.4) |  |
|  | missing | 13 | 6 | 19 |  |
| Other | no | 220 (95.7) | 229 (97.4) | 449 (96.6) | 0.319 |
|  | yes | 10 ( 4.3) | 6 ( 2.6) | 16 ( 3.4) |  |
|  | missing | 13 | 7 | 20 |  |
| Not specified | no | 230 (99.6) | 236 ( 100) | 466 (99.8) | 0.495 |
|  | yes | 1 ( 0.4) | 0 ( 0.0) | 1 ( 0.2) |  |
|  | missing | 12 | 6 | 18 |  |
