# Supplementary figures and images for: Patient-reported outcomes for the Intergroup Sentinel Mamma study (INSEMA): A randomised trial with persistent impact of axillary surgery on arm and breast symptoms in patients with early breast cancer
Source: eClinicalMedicine. 2022 Nov 25;55:101756. doi: 10.1016/j.eclinm.2022.101756 (PMC9706517; doi:10.1016/j.eclinm.2022.101756)

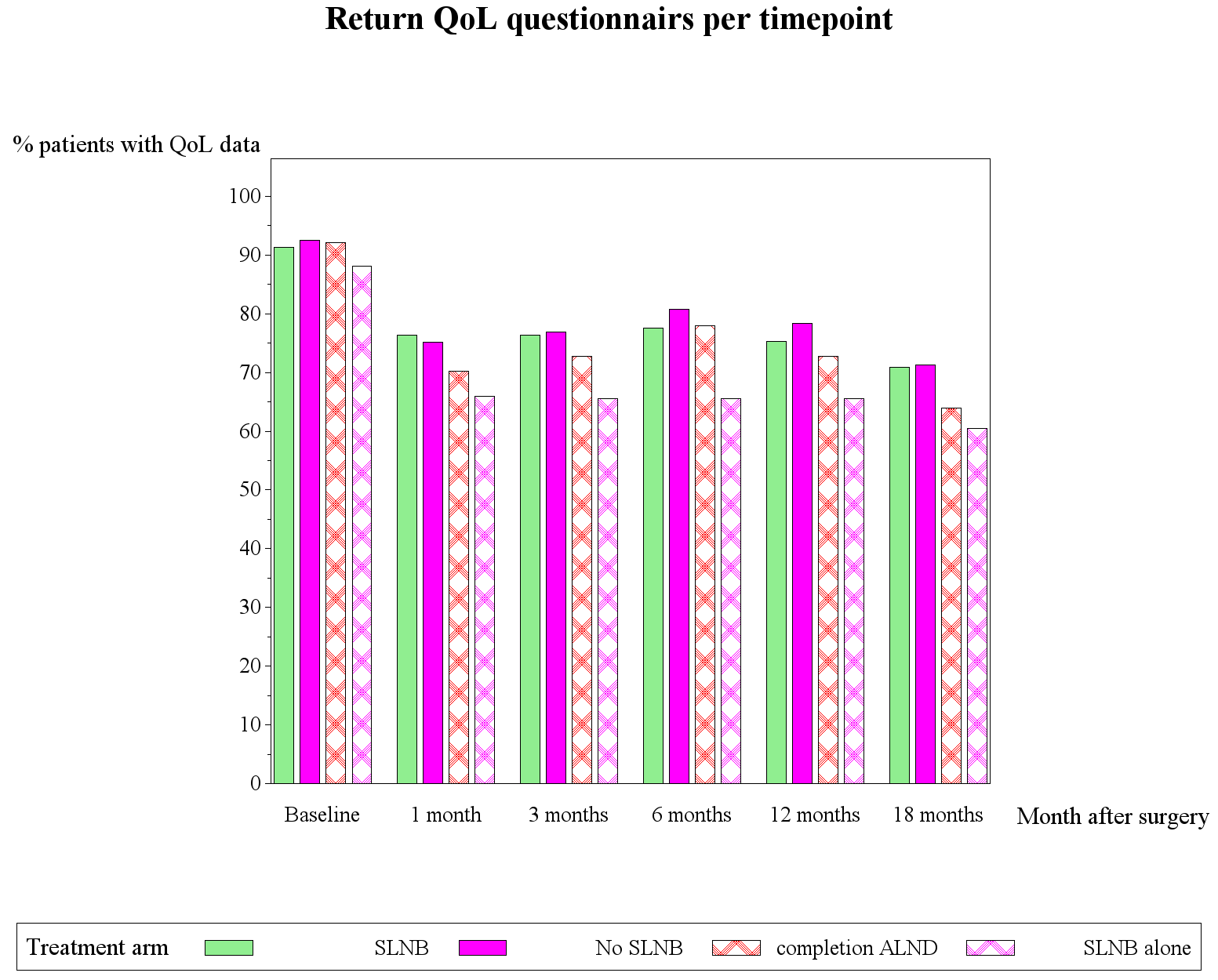

Supplement: Supplementary Figure S1 — Questionnaire response rates per follow-up timepoint with respect to the treatment arms. [file mmc3.docx]

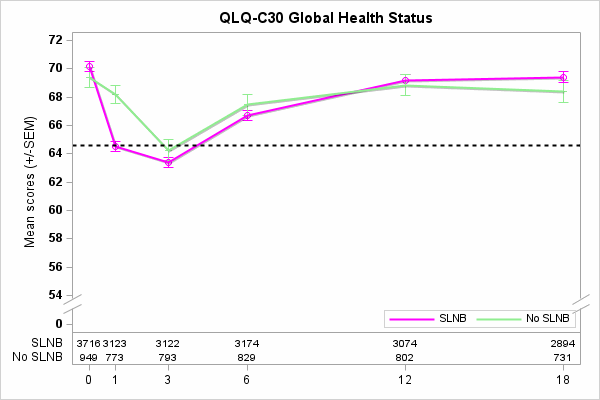


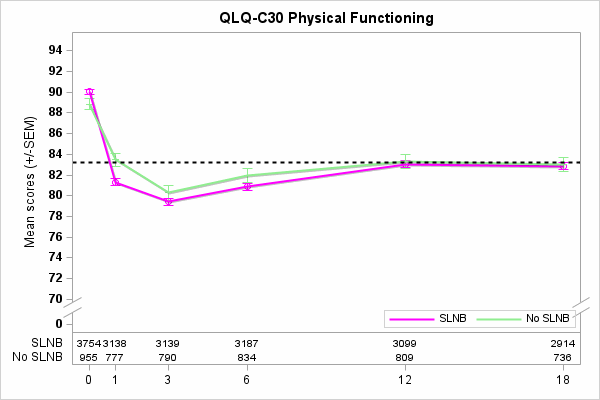


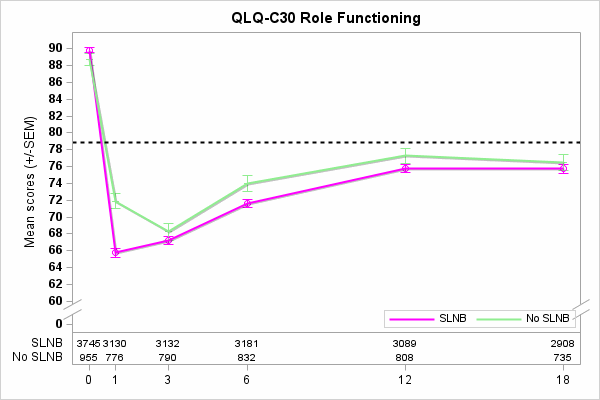


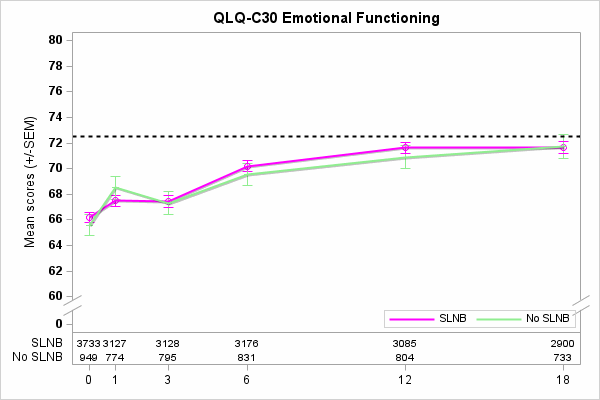


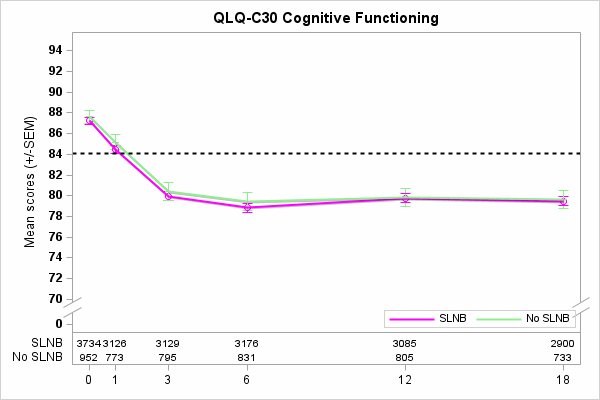


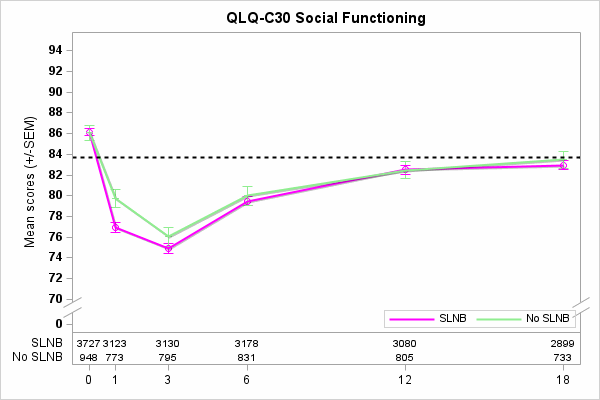


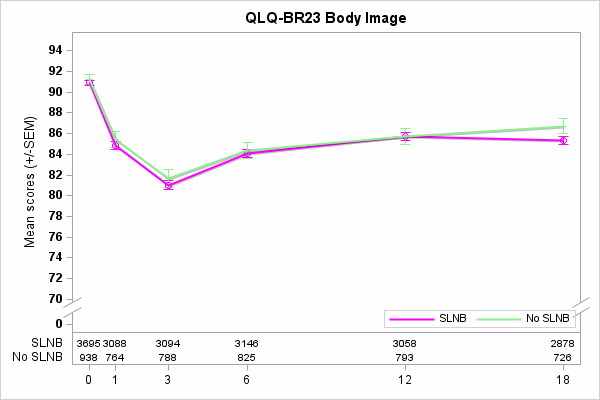

Supplement: Supplementary Figure S2 — Curves for PROs concerning QLQ-C30 and QLQ-BR23 questionnaires and first randomization. According to a reference manual, reference lines are for stage I-II breast cancer, available only for QLQ-C30, not for QLQ-BR23 Body Image. A: Global Health Status; B: Physical Functioning; C: Role Functioning; D: Emotional Functioning; E: Cognitive Functioning; F: Social Functioning; and G: Body Image. [file mmc4.docx]

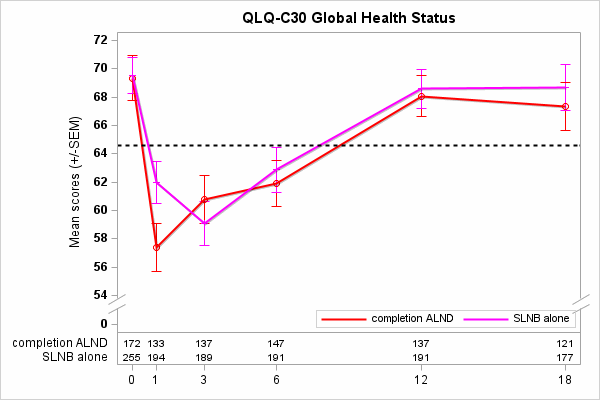


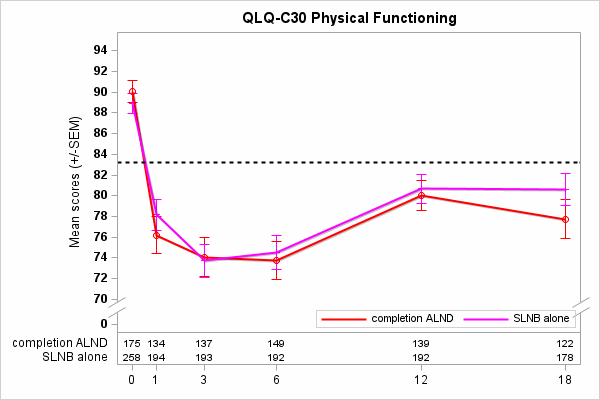


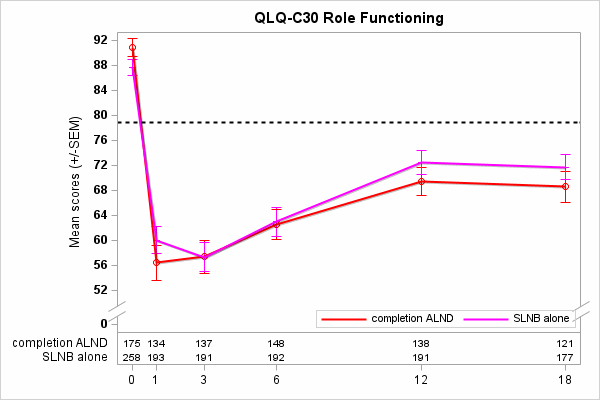


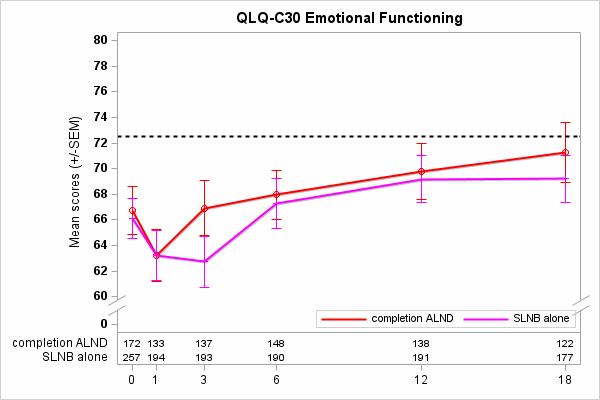


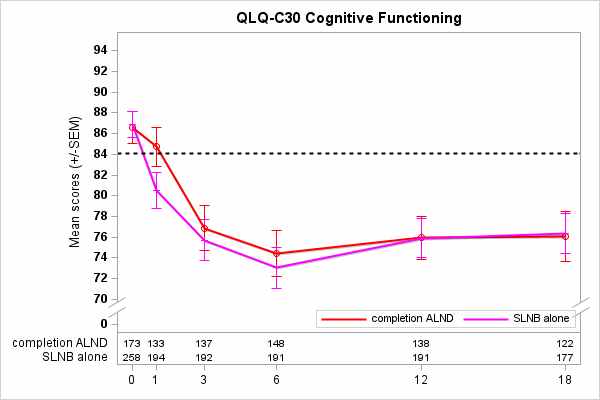


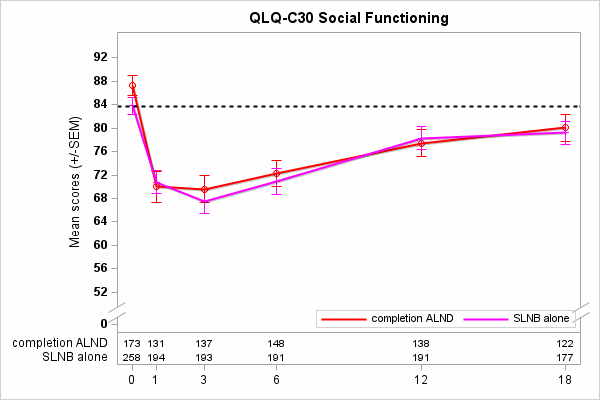


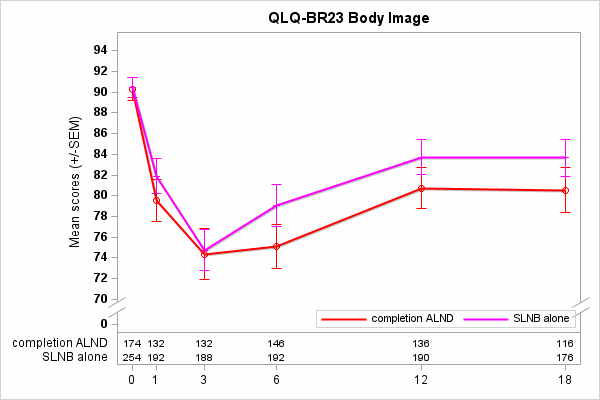

Supplement: Supplementary Figure S3 — Curves for PROs concerning QLQ-C30 and QLQ-BR23 questionnaires and second randomization. According to a reference manual, reference lines are for stage I-II breast cancer, available only for QLQ-C30, not for QLQ-BR23 Body Image. A: Global Health Status; B: Physical Functioning; C: Role Functioning; D: Emotional Functioning; E: Cognitive Functioning; F: Social Functioning; and G: Body Image. [file mmc5.docx]
